# Machine Learning Based Fault Classification in Pilot Plant Batch Reactor: Using Support Vector Machine

**DOI:** 10.1021/acsomega.4c04421

**Published:** 2024-06-19

**Authors:** Arockiaraj Simiyon, Chaitanya Sachidanand, Manthana Halmakki Krishnamurthy, Ananya V. Bhatt, Thirunavukkarasu Indiran

**Affiliations:** † Manipal School of Information Sciences, Manipal Academy of Higher Education, Manipal 576 104, India; ‡ Department of Instrumentation and Control Engineering, Manipal Institute of Technology, Manipal Academy of Higher Education, Manipal 576 104, India

## Abstract

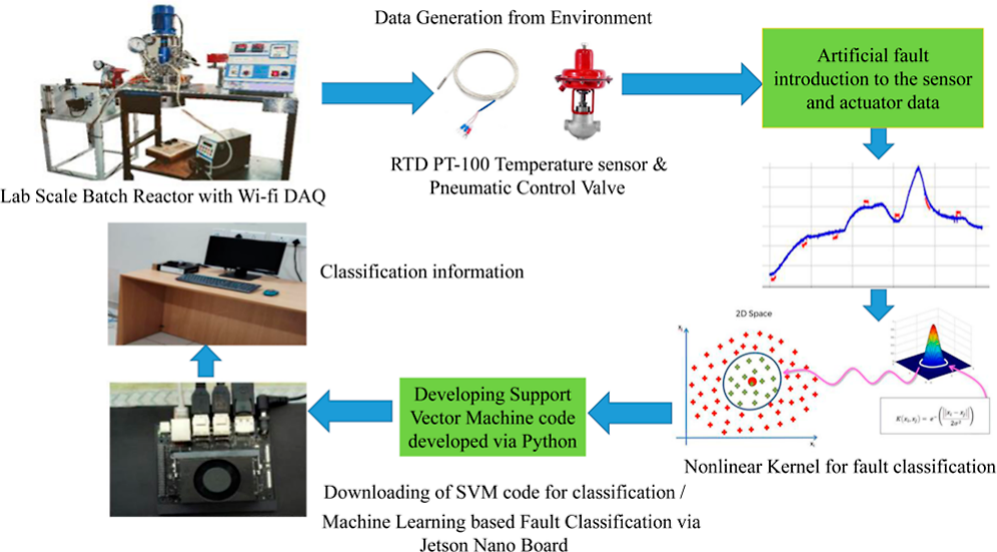

Identifying and diagnosing faults is a critical task in process industries to maintain effective monitoring of process and plant safety. Minimizing process downtime is critical for enhancing the quality of the product and minimizing production costs. Real-time categorization of issues across several levels is essential for the monitoring of processes. However, there are still notable obstacles, that must be addressed, such as the existence of robust correlations, the complexity of the data, and the lack of linearity. This study introduces a novel fault identification technique in batch reactor experimental trials that employs multikernel support vector machines (SVMs) to categorize internal and external issues, specifically reactor temperature, coolant temperature, and jacket temperature. The data set was obtained from empirical research. The classification has been conducted using a multikernel SVM. This article identified that the nonlinear classifier using the radial bias function results in an accuracy that is at least 22.08% superior to other methods.

## 1 Introduction

A batch reactor (BR) is a type of chemical reactor in which a process occurs in discrete batches rather than in a continuous stream. In this system, a finite quantity of reactants is introduced into the reactor, allowed to undergo the desired chemical transformation, and then the product is removed before a new batch begins. Unlike continuous reactors, BRs offer greater flexibility in handling various chemical processes and are particularly useful for small-scale production or when the reaction parameters need to be closely controlled. The sequential nature of batch processing allows for better monitoring and adjustment of reaction conditions, making it appropriate for a wide range of applications in industries such as pharmaceuticals, specialty chemicals, and food processing. BRs are employed when precise control, product quality, or the need for frequent changes in production parameters are critical to the success of the chemical process.^1^

**Figure 1:**
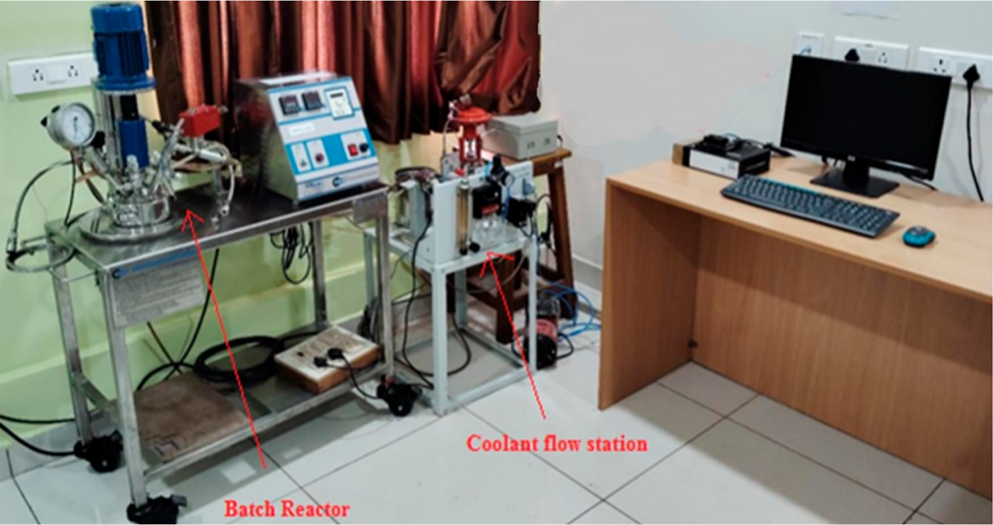
Pilot plant BR setup with Jetson Orin 8GB board available in “Machine Learning for Advanced Process Control lab”, MIT, Manipal. Image captured by Thirunavukkarasu Indiran.

**Figure 2:**
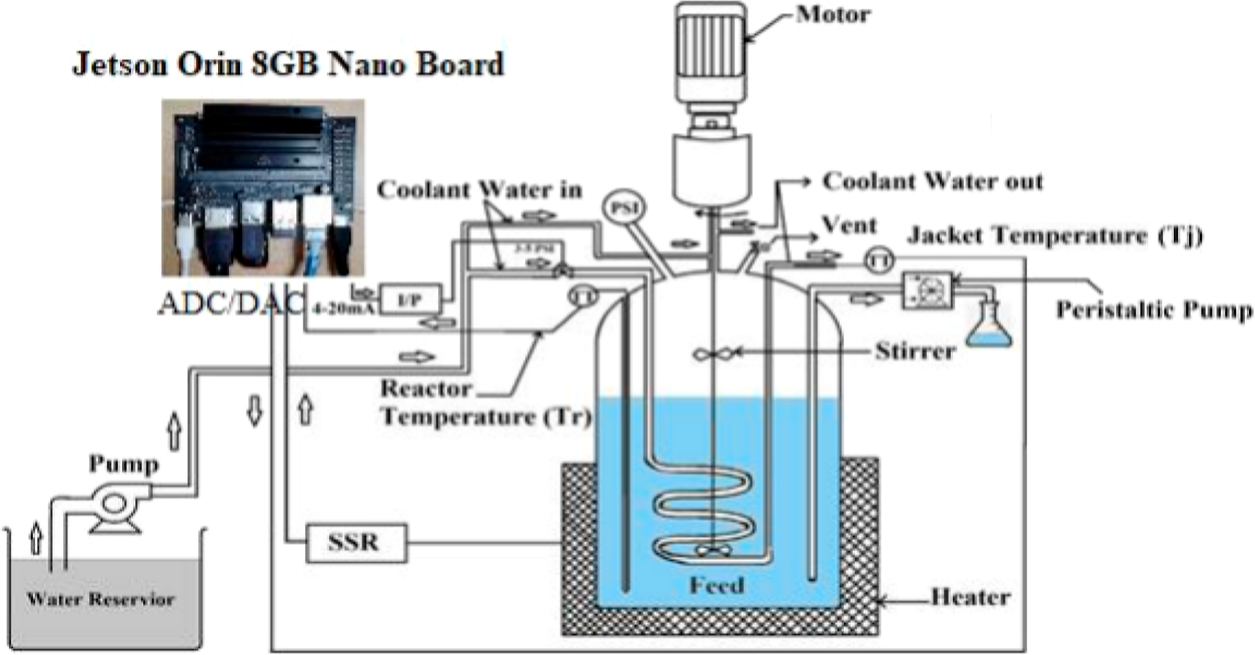
Schematic of lab-scale BR view.^30^

BR processes are commonly used in the pharmaceutical and chemical industries for the production of various pharmaceuticals, chemicals, and specialty products. However, these processes are susceptible to various faults and anomalies that can lead to undesirable outcomes, such as decreased yield, product quality deviations, or even hazardous conditions. Early detection and classification of these faults are essential for timely intervention and effective process control. The data from the BR, shown in Figure 1, was used for this purpose. Figure 2 shows the schematic of the BR setup. It included the value of the actuator (*F*_c_), the temperature of the coolant (*T*_c_), the temperature of the reactor (*T*_r_), and the temperature of the jacket (*T*_j_). The most robust machine learning (ML) method for nonlinear data classification, called support vector machines (SVMs), has been used for the classification of faults in intricate industrial processes.^2^

The SVMs are highly efficient in handling high-dimensional and noisy data, as well as scenarios where the data may not exhibit linear separability. Traditional SVMs are designed to work with linearly separable data, but they can struggle with complex, nonlinear relationships. To address this limitation, researchers have developed the concept of using multiple kernels in SVMs, known as the multiple kernel SVM. This approach’s ability to handle nonlinearity can lead to enhanced process understanding, early fault detection (FD), and improved process control, ultimately contributing to safer and more efficient industrial operation.^3^

The data collected from the BR is nonlinear in nature. In closed-loop experiments, such as BR setups, where events are tightly controlled and interconnected, the applicability of the methods used in ref (4) may be limited. This is because these techniques are primarily designed for open loop experiments where events occur more independently and unpredictably. The most robust ML method for nonlinear data classification is multikernel SVM. Therefore, the classification of faults in intricate industrial processes has utilized this approach.

This paper optimized the radial bias function kernel and key hyperparameters such as C, γ, and decision_function_shape by using GridSearchCV to do a robust parameter grid search. This meticulous selection process enabled us to identify the optimal combination that significantly improved the model’s performance. The proposed multikernel SVM approach for FD in the BR system improved accuracy on nonlinear data. This made the proposed method different from other methods that are already available.

The primary advantage of this approach lies in its ability to effectively handle complex nonlinear relationships within the data, leading to more accurate FD compared to traditional single-kernel methods. The parameter tuning ensured that the model achieved the best possible generalization and detection capability. This showcases the superiority of the multikernel SVM in handling intricate FD scenarios in BR processes.

## 2 Literature Review

In the literature study, the authors have included additional studies related to process safety and environmental protection that focus on advanced methods of FD and diagnosis from a safety standpoint, in addition to those listed in the article.

Jiang et al.^4^ proposed hybrid FD and diagnosis approaches. This approach, integrated with safety and risk assessment (RA), is being explored in various domains, including nuclear power plants. Techniques like dynamic dependency event trees (DDET), Markov/continuous time Markov chain (CCMT), and GO-FLOW are utilized in these endeavors. In a nuclear power plant scenario, potential failures such as radiation leaks, material decay, or sensor malfunctions can occur due to a multitude of factors. These failures often cascade, leading to a series of interconnected events. DDET essentially creates a roadmap of potential accidents, illustrating how various events could unfold over time as conditions evolve. Markov/CCMT techniques involve analyzing the interactions between different systems within the plant to understand failure probabilities based on historical data. GO-FLOW focuses on emergency planning, helping to develop efficient strategies for managing crises effectively. However, in closed-loop experiments like BR setups, where events are tightly controlled and interconnected, the applicability of the methods used in ref (4) might be limited. This is because these techniques are primarily designed for open-loop experiments where events occur more independently and unpredictably.

Amin et al.^5^ utilized the Naïve Bayes classifier (NBC) in this study of FD and dynamic RA. It calculates posterior probabilities for fault classes based on process data, aiding in fault diagnosis and predicting failure risks. The NBC’s assumption of feature independence simplifies calculations, and advanced versions can handle feature dependencies for improved accuracy. Overall, the NBC is chosen for its efficiency, simplicity, and effectiveness in generating fault class probabilities for RA in chemical engineering processes.

While the long short-term memory (LSTM) neural network used in Tamascelli et al.^6^ has its advantages for sequence prediction tasks, it also has some drawbacks compared to SVMs in certain contexts such as interpretability, training time, data efficiency, hyperparameter tuning, and handling sequential data. SVM models are generally easier to interpret and understand compared to complex neural networks like LSTMs. SVMs provide clear decision boundaries, making it easier to understand how the model makes predictions. In contrast, LSTMs are more complex, and their inner workings may be harder to interpret. SVMs are known for their relatively fast training times, especially when dealing with small to medium-sized data sets. On the other hand, training deep neural networks like LSTMs can be computationally intensive and time-consuming, particularly with large data sets. SVMs are effective with small to medium-sized data sets and can perform well with limited training data. In contrast, LSTMs, being deep learning models, may require larger amounts of data for training to generalize well and avoid overfitting. SVMs have fewer hyperparameters to tune compared to neural networks like LSTMs. Tuning the hyperparameters of LSTMs can be a more complex and time-consuming process.

In this paper, Wang et al.^7^ employed the context of drilling and production platforms and ML for tasks like real-time data monitoring and analysis to enhance drilling efficiency, minimize downtime, and improve safety. ML algorithms are used for predictive maintenance by analyzing sensor data to predict failures of the equipment before they occur. This method allows for the proactive scheduling of maintenance and decreases both downtime and maintenance expenses. The approach in this paper involves leveraging ML algorithms and big data analytics to optimize drilling operations, predict key parameters, mitigate risks, and improve overall efficiency and safety in the oil and gas industry. Since our experiment is closed loop and the data generated is not highly varying, a big data approach cannot be applied.

In this paper, El-Kady et al.^8^ employed various data science techniques to optimize offshore wind-integrated hydrogen production systems, enhance efficiency, and mitigate risks. This paper includes some key techniques such as geospatial modeling, which utilizes geospatial methods to estimate the levelized cost of hydrogen production; ML techniques to predict the wind speed, which is essential for optimizing energy production; data analysis methods to analyze vast amounts of data in real-time, providing actionable insights for decision-making; and asset management techniques using unsupervised ML for life-extension classification of offshore wind assets, aiding in asset management and maintenance planning. A few of the fundamental ideas for risk modeling were considered in this paper on how the data could vary if errors occur and data modeling using SVM is performed instead of the technique specified in this paper since it was well suited for our data.

The research by Liu et al.^9^ employs a data-driven scheme for early prediction of abnormal situations in chemical processes. The methodology involves integrating K-means clustering and density-based spatial clustering of applications with noise algorithms with bidirectional long short-term memory and multilayer perceptron models to enhance the effectiveness of the warning techniques. This study emphasizes the significance of combining these data-driven fusion algorithms to improve the accuracy and scientific validity of abnormal condition prediction in chemical processes. By leveraging these techniques, the research aims to provide a more robust early warning system for identifying potential issues in industrial settings, thereby enhancing process safety and efficiency.

Zhang et al.^10^ presents a novel wavelet transform that incorporates an enhanced technique for selecting the appropriate wavelet basis function. Here, the most suitable wavelet basis function is chosen by evaluating its distance and mean values. The features obtained from this optimal wavelet basis function are regarded as the most effective characteristics for signals. The attributes are randomly and evenly divided into training data and testing data. A diagnosis model based on a “multiple kernel extreme learning machine (MKELM)” is started using the training data. The parameters of MKELM are determined using the particle swarm optimization approach. Ultimately, MKELM is employed to detect defects in the testing data to validate its performance. The proposed MKELM outperforms the backpropagation neural network, SVM, and ELM classifiers, as well as deep learning algorithms, in terms of diagnostic accuracy.

Cheny et al.^11^ presents a “curvilinear distance metric learning (CDML)” technique that dynamically learns the nonlinear geometries of the training data. The CDML is parametrized by a 3-order tensor, as stated by Weierstrass theorem. The optimization approach is specifically built to learn the tensor parameter. Theoretical analysis is conducted to ensure the efficacy and validity of CDML. This method has been extensively tested on both synthetic and real-world data sets, and the results confirm that it outperforms other metric learning models currently available.

Zhang et al.^12^ introduces a new method for identifying initial faults in Analog circuits. The time responses are obtained by sampling the outputs of the circuits being tested. These responses are then decomposed using the wavelet transform to yield energy characteristics. Subsequently, the kernel entropy component analysis generates lower-dimensional features, which are then used as samples for training and testing a one-against-one least-squares SVM. The simulations of the initial fault diagnosis for a Sallen-Key band-pass filter and a two-stage four-op-amp biquad low-pass filter illustrate the diagnostic procedure of the suggested methodology. Additionally, they indicate that the proposed approach achieves higher diagnostic accuracy compared to the referenced methods.

Elgamasy et al.^13^ presents a fast FD method for voltage source converter-based high-voltage direct current (HVDC) transmission systems. Bergeron model equations determine an adopted transmission system’s remote terminal voltage. These equations consider local current and voltage signals. The computed remote terminal voltage is then compared to the measured and transmitted value. Calculated and observed voltages are almost identical if the transmission system is working properly. However, faults cause large virtual voltage. A voltage differential above a threshold indicates a failure. A reliable communication method is needed, but communication time does not delay defect identification. For system evaluation, PSCAD/EMTDC creates a thorough simulation. The simulation incorporates faults near the protected transmission system’s interior or exterior. The results show that a modest sample and processing frequency quick detection method works. Even with serious exterior defects or mismatched terminal samples, security is strong. This supports the multiterminal HVDC network protection scheme.

The studies do not clearly explain the effectiveness of the proposed ML algorithms in capturing intricate nonlinear relationships in data and addressing nonlinear dependencies among variables in FD and RA situations. The proposed approach to chemical engineering processes selects SVMs for FD and RA because of their capability to capture intricate nonlinear correlations in data and manage nonlinear dependencies among variables. Factors influencing this choice may include simplicity, efficiency, interpretability, and meeting specific requirements for FD and dynamic RA.

## 3 Methodology

SVMs are a reliable type of supervised ML algorithm designed for classification and regression applications. SVMs fundamentally seek to identify the most ideal hyperplane that effectively distinguishes various classes within a certain data set. SVMs distinguish themselves by their capability to manage intricate decision boundaries and nonlinear associations by employing kernel functions. This methodology seeks to maximize the margin, defined as the spatial gap between the hyperplane and the closest data points from each class. This attribute not only improves the model’s ability to apply to fresh data but also ensures its resilience when dealing with data sets that are noisy or have overlapping elements. SVMs find widespread application in various domains such as text classification, image recognition, and bioinformatics. This is due to the adaptability and efficacy of SVMs in handling intricate and multidimensional data. It is particularly well-suited for classification problems and is widely recognized for its ability to handle both linear and nonlinear data.^14^

### 3.1 Classification in SVM

SVMs can be used for both linear and nonlinear classifications of data into various categories. Section 3.1.1 discusses the significance of a binary classification using a linear SVM, and Section 3.1.2 highlights the uses of a nonlinear SVM and how that can be extended to handle data with multiple classes for classification through various strategies.

#### 3.1.1 Binary Classification

In order to partition a data set into two distinct classes, binary classification with SVMs seeks to find the optimal hyperplane that maximizes the distance between the classes. A hyperplane is a boundary that separates instances belonging to different classes. SVMs strive to find the distance between the proximal data points of each class, while also accurately classifying the data. To achieve this, SVMs utilize the data points that are in closest proximity to the decision border, called support vectors. By positioning the ideal hyperplane to maximize the margin, generalization to new, unknown data is enhanced. SVMs utilize kernel functions to transform the input data into a higher-dimensional space when linear separation is not possible.^15^

The decision function of an SVM assigns new instances to one of the two classes based on their position relative to the hyperplane. SVMs are particularly effective in scenarios with well-defined class boundaries and are widely utilized in various applications, including spam detection, image recognition, and medical diagnosis, where binary classification is a common requirement.^16^

#### 3.1.2 Multiclass Classification

SVMs can be extended to handle data with multiple classes for classification using through various strategies. One common approach is the one-versus-all (OvA) or one-versus-the-rest (OvR) method. In this strategy, for each class, a binary classifier is trained to distinguish that class from the rest of the classes. Once these binary classifiers are trained, the class with the highest decision function output becomes the predicted class.^17^

Another approach is the one-versus-one (OvO) method, where a binary classifier is trained for every pair of classes. In this case, the number of binary classifiers is obtained from eq 1.
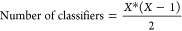
1Here, *X* is the number of classes. During the prediction process, the class that receives the most votes from each classifier determines the final predicted class. SVM inherently supports binary classification, and these strategies provide a way to extend them to handle multiclass scenarios. Frequently, the choice between OvA and OvO is contingent on the nature of the problem, the extent of the data set, and computational factors. SVMs remain a popular choice for multiclass classification due to their ability to handle higher-dimensional data and find complex decision boundaries across multiple classes.^18^

### 3.2 Deciding Factors in SVM

**Figure 3:**
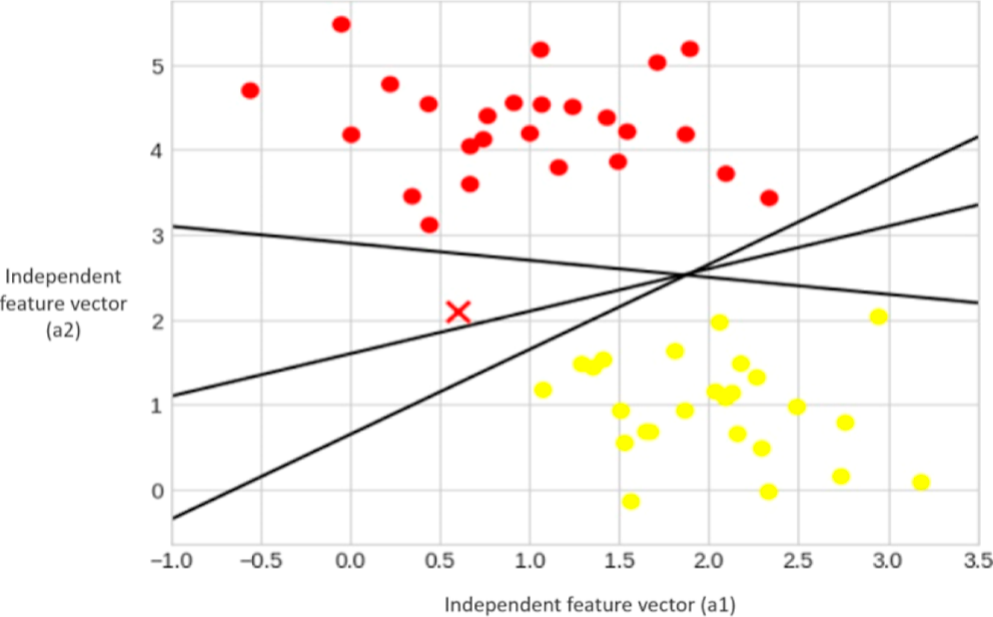
Three perfect linear discriminative classifiers plotted for the classification of two classes.

**Figure 4:**
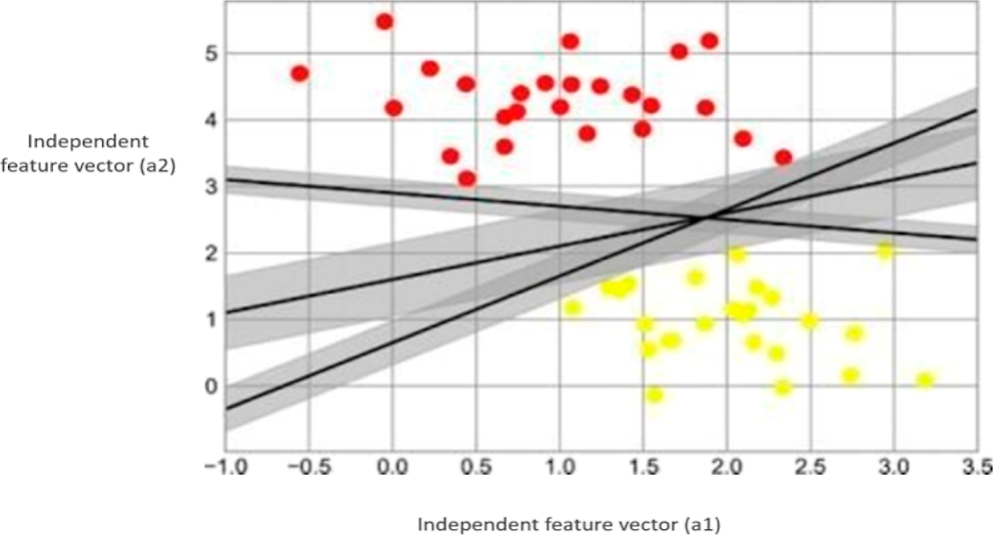
Visualization of “margins” within discriminative classifiers plotted for the classification of two classes.

**Figure 5:**
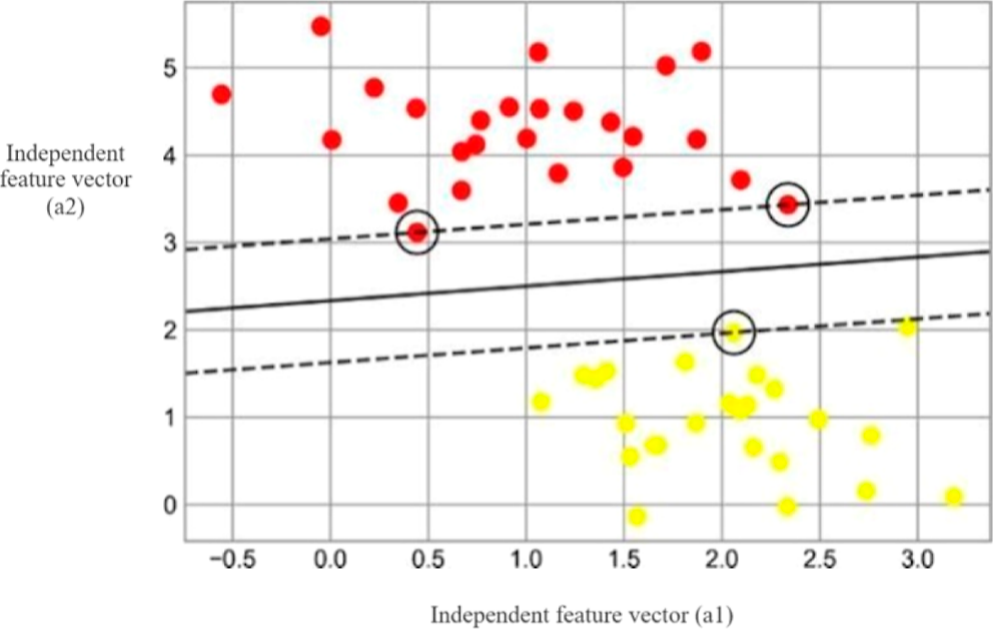
SVM classifier fits the data with margins (dashed lines) and support vectors (circles) plotted for the classification of two classes.

- **Hyperplane:** the fundamental principle of SVM revolves around the notion of a hyperplane, which is a multidimensional surface that effectively distinguishes data points belonging to different classes. In a two-dimensional space, a hyperplane can be defined as a straight line; however, in higher dimensions, it is referred to as a hyperplane. In Figure 3, three separate hyperplanes can be employed for the classification of two classes. In the scatter plot, the *X*-axis corresponds to the values of the independent feature vector (a1), while the *Y*-axis corresponds to the values of the independent feature vector (a2).^19^
- **Margin:** the primary goal of SVM is to determine the hyperplane that optimizes the margin, defined as the maximum distance between the hyperplane and the nearest data points belonging to different classes. This enhances the model’s ability to generalize to unfamiliar data. In Figure 4, a shaded area delineates the margin that corresponds to each hyperplane. In the scatter plot, the *X*-axis corresponds to the values of the independent feature vector (a1), while the *Y*-axis corresponds to the values of the independent feature vector (a2).^20^
- **Support vectors:** the data points that are located closest to the hyperplane and have the most significant impact on its position. These points provide evidence for the decision limit. The data points identified as support vectors are encircled, as shown in Figure 5. In the scatter plot, the *X*-axis corresponds to the values of the independent feature vector (a1), while the *Y*-axis corresponds to the values of the independent feature vector (a2).^21^
- **Kernel trick:** SVM is able to effectively deal with nonlinear data by utilizing the kernel technique. SVM utilizes this method to automatically convert the data into higher dimensions, enabling the effective separation of classes using a linear hyperplane. Typical kernel functions comprise the linear kernel, the polynomial kernel, and the RBF.^21^
- ***C* parameter**: the SVM algorithm includes a regularization parameter called “*C*”. It manages the balance between increasing the margin and decreasing the categorization mistake. A lower value of *C* encourages a wider margin, which can lead to a higher number of misclassifications. On the other hand, a larger value of *C* leads to a smaller margin and fewer misclassifications. SVMs provide a means to enhance this. Instead of just creating a line with no width to separate the classes, we can add a margin of a certain width around each line, extending up to the nearest point.^21^
- **Gamma parameter**: it determines the impact of each individual training example or data point on the decision boundary. More precisely, it determines the extent to which a single training example impacts the overall outcome. A small γ means that the influence is more extensive, leading to a smoother decision boundary, and the model might generalize well but may be prone to overfitting. Conversely, a large γ results in a more localized influence, creating a decision boundary that tends to learn the training data more correctly but may not generalize well to unseen data, potentially causing overfitting.^22^

### 3.3 Weight Calculation for Two Class SVM

In SVMs, the weights, denoted as *w*, are an important part of the model. The decision boundary is the locus of points where the inner product of the feature vector *x* and weight (*w*) added with a bias (*b*) equals zero, as given in eq 2. In this context, the weight vector is denoted by “*w*”, the input feature vector is represented by “*x*”, and the bias term is indicated by “*b*”. The weight vector *w* can be represented as the sum of the support vectors multiplied by their corresponding coefficients^23^
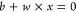
2

Equation 3 is used to calculate the *f*(*x*) values. Vectors with negative and positive *f*(*x*) values are categorized into distinct classes. The decision function *f*(*x*) assigns a signed distance from the hyperplane to each sample, and predictions are determined by the sign of this distance. Positive values indicate one class, while negative values denote the other.^24^
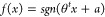
3

In eq 3, θ^*t*^·*x* is the dot product between the transpose of the *d*-dimensional vector θ^*t*^ and the input feature vector *x*. *a* is the bias term, a constant that shifts the decision boundary. It allows the decision boundary to be independent of the origin and controls the offset from the origin. To acquire the maximum margin, the width maximization outlined in eq 4 is used. The degree of misclassification can be measured by the slack variable ε_*i*_, where *P* is the error penalty. The solution for the maximum margin can be obtained by solving the minimization problem given by minimize.^25^

To acquire the maximum margin, the width maximization outlined in eq 4 is used.
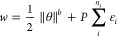
4

### 3.4 Weight Calculation for Multikernel SVM

To solve the constrained optimization issue, one can utilize a Lagrange multiplier λ_*i*_, with the condition that λ_*i*_ is greater than or equal to zero (λ_*i*_ ≥ 0). This corresponds to the limitation imposed on a support vector. The solution can be expressed as the minimization of weight as given in eq 5.^26^
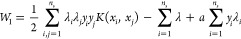
5

In the context of the dual optimization problem for SVMs, the indices *i* and *j* represent the individual training samples in the data set. The Lagrange multipliers (λ_*i*_ and λ_*j*_) are associated with each training sample in the data set. For a data set with *N* samples, there are *N* Lagrange multipliers. The optimization problem involves finding the values of these λ_*i*_ coefficients. *y*_*i*_ and *y*_*j*_ are the class labels associated with the training samples *x*_*i*_ and *x*_*j*_. The kernel function is a crucial concept in SVMs, especially when dealing with nonlinearly separable data. It allows the SVM to implicitly operate in a higher-dimensional feature space without explicitly computing the transformation. *K*(*x*_*i*_,*x*_*j*_) is the kernel function, which computes the similarity or distance between the input vector *x* and the *i*-th support vector *x*_*j*_. The value of offset “*a*” is as follows
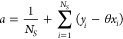
6Here, *N*_s_ is the total number of samples used for training the SVM model. It is a constant that shifts the decision boundary away from the origin in the feature space. It allows the SVM to capture the offset or translation of the decision boundary, making the SVM more flexible in handling data that may not be perfectly separable by a hyperplane passing through the origin.^27^

The Lagrange multipliers, commonly referred to as dual coefficients, have a pivotal role in the design and optimization of SVMs. The SVM is a quadratic optimization method that involves constraints. It is commonly addressed by employing the Lagrange duality technique. The optimization issue in eq 5 can be formulated as the objective of minimizing *W*_2_ as given in eq 7.
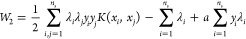
7

In the context of SVMs, the dual coefficients attribute contains the coefficients associated with the support vectors in the dual problem formulation. In the case of multiclass classification, there is a set of dual coefficients for each class against the rest. For a binary classification problem, dual_coefficient is a 1D array of shape (n_support_vectors). For a multiclass problem, it is a 2D array of shape (*n*_classes – 1, *n*_support_vectors). Table 1 shows the parameters used in optimization problem.^28^

**Table 1:** Parameters Used in Optimization Problem

symbols	parameters	values
*W*_1_, *W*_2_	weight vectors	initialized with 0
λ	Lagrange multiplier	array of values ranges between 0.5 and 1. Number of values of the array depends on the data set and the selected kernel
θ	D-dimensional vector	determined from the data set
*K*(*x*_*i*_,*x*_*j*_)	Kernel function	Gaussian function determines the values
*N*_s_	total number of samples	123,756 samples including faulty data
*P*	error penalty	initialized with 0
ε	Slack variable	initialized with 0
*b*	bias	initialized with 0

When initializing an SVM with an RBF kernel, the weight vector, slack variable, bias, and error penalty variables are all set to zero. However, during the optimization process, these values will be iteratively modified to minimize the objective function. The objective function tries to maximize the margin while simultaneously decreasing the classification error. Zero serves as the initial point from which the method proceeds to iterate in order to discover the optimal values.

#### 3.4.1 Parameter Setting Scheme

Weight vectors (*W*_1_, *W*_2_) are initialized with zero. During training, the SVM model updates and finds the optimal weight vectors. The optimal weights are those that provide the maximum margin between classes while minimizing classification error. The Lagrange multiplier (λ) values are not directly set when initializing the parameters. The number of values in the array depends on the data set and the selected kernel. The optimization process internally adjusts these values to meet the constraints of the model. The *sklearn* library has algorithms like sequential minimal optimization to solve for the optimal Lagrange multipliers. The final calculated values for (λ) are an array of values ranging between 0.5 and 1. The D-dimensional vector (θ) is calculated from the data set and represents the transformed feature space using the RBF kernel. The RBF kernel function computes the exact values during this implicit transformation on the data set.^29^

In the Gaussian (RBF) kernel *K*(*x*_*i*_,*x*_*j*_), kernel parameters are mainly controlled by the hyperparameter γ. The default value for γ in *sklearn* is “*scale*”, which is . This parameter is tuned during cross-validation.

The *C parameter* in *sklearn* controls the *error penalty* (*P*), balancing the tradeoff between a smooth decision boundary and correctly classifying the training points. A higher *C* value aims for correct classification of all training examples, while a lower *C* allows for a wider margin. This can be adjusted during cross-validation. *Slack variables* (ε) are implicitly managed in *sklearn* through the *C parameter*. The *C parameter* controls the allowance for misclassifications (slack) in the model. The *bias* (*b*) is also adjusted during the training process. The *sklearn* SVM classifier computes this as part of the optimization process to properly position the decision boundary. The *error penalty* (*P*), *slack variables* (ε), and the *bias* (*b*) values can be initialized with 0.^29^

#### 3.4.2 Adjustment Process in *Sklearn*

Initially, set the parameters to the values given in Table 1, and then train the model using the *sklearn fit* method. The *fit* method allows the model to learn from the training data. This method uses the internal optimization algorithm to adjust the Lagrange multiplier (λ) values, weight vectors, bias, and other parameters. The *RBF kernel* function transforms the input data into a higher-dimensional space where linear separation is possible. Finally, the optimization process involves solving a quadratic programing problem to find the support vectors, which implicitly define the weight vectors and bias. The hyperparameters, such as *C* and γ, are fine-tuned using techniques like grid search with cross-validation to find the optimal values that result in the best performance.^29^

In this example, dual_coefficients_per_class will be a 2D array, where each row corresponds to the dual coefficients for a specific class. The number of rows will be *n*_classes – 1, where *n*_classes is the total number of classes.^29^

#### 3.4.3 Weights (*W*_1_, *W*_2_)

When SVMs are used for nonlinear classification tasks, the input data is mapped into a higher-dimensional space where the classes become linearly separable. In this higher-dimensional space, each feature corresponds to a dimension, and the weight vector (*w*) represents the coefficients of the hyperplane that separates the classes. In the context of nonlinear data classification, the weight vectors capture the relationship between the input features in the higher-dimensional space. These weight vectors are learned during the training process, where the SVM algorithm optimizes them to find the hyperplane that maximizes the margin between classes. The RBF kernel, commonly used for nonlinear SVM classification, implicitly maps the input data into a higher-dimensional space using a Gaussian-like function. In this space, the SVM learns the optimal weight vectors that define the decision boundary. These weight vectors determine the influence of each feature in the decision-making process, allowing the SVM to capture complex relationships between the input features and accurately classify nonlinear data.^29^

#### 3.4.4 λ (Lagrange Multiplier)

In the context of SVM, λ usually represents the regularization parameter. In scikit-learn’s SVM implementation, it is denoted as “*C*” in the parameters_dictionary. It controls the tradeoff between achieving a low training error and a low testing error due to overfitting. Higher values of λ result in stronger regularization.^29^

#### 3.4.5 *D*-Dimensional Vector (Θ)

The value for a *D*-dimensional vector (*x*) in SVMs depends entirely on the data set and the features used to represent each data point. In an SVM context, each data point is represented as a vector in a *D*-dimensional feature space, where *D* is the number of features used to describe the data. These features could be numerical values, categorical variables, or even derived features based on some transformation of the original data. For example, if you are working with a data set of house prices and you are using features such as square footage, number of bedrooms, and number of bathrooms, then each data point would be represented as a 3-dimensional vector (*x*_1_, *x*_2_, *x*_3_) in the feature space. The values of these features are determined by the data set itself. So, for a given data set, the value for each element of the *D*-dimensional vector (*x*) would be determined by the specific characteristics of the data points being represented.^29^

#### 3.4.6 Bias (*b*)

The bias in SVM, often denoted as *b*. In the context of SVM, *b* is not directly bounded to a specific numerical range because its value is determined during the optimization process, aimed at maximizing the margin between classes.^29^

However, the bias term *b* is not completely unconstrained. Its value is adjusted during training to ensure the optimal hyperplane that separates the classes. So, while there’s no predetermined numerical range for *b*, its value is determined by the optimization algorithm and the specific data set being used.^29^

#### 3.4.7 Slack Variable (ε)

In the context of SVM, a slack variable is introduced to handle the case when the data is not linearly separable. Slack variables allow some training examples to be misclassified or fall within the margin, thus relaxing the strict margin requirement of hard-margin SVMs. In the optimization problem for SVMs, slack variables are denoted typically by ε_*i*_. Each ε_*i*_ corresponds to a training example, and its value represents how much the corresponding example violates the margin or the hyperplane’s boundary. The introduction of slack variables leads to a modified optimization problem, where the goal is to minimize a combination of the margin size and the amount of slack used, often subject to some regularization term. This modified optimization problem is usually solved using methods like quadratic programing. Slack variables play a crucial role in soft-margin SVMs, allowing them to handle nonlinearly separable data by finding a balance between maximizing the margin and minimizing the classification error.^29^

#### 3.4.8 Error Penalty (*P*)

In the context of SVM, the error penalty refers to the cost associated with misclassifying data points. When SVMs are used for classification tasks, they aim to find a hyperplane that separates different classes with a maximum margin while minimizing the misclassification error. However, in real-world scenarios, perfect separation may not be achievable, especially when the data is not linearly separable. In such cases, SVMs use soft-margin classification, which allows for some misclassification to find a better overall solution. The error penalty in SVMs is typically controlled by a regularization parameter, often denoted as *C*. This parameter determines the tradeoff between maximizing the margin and minimizing the classification error. A smaller value of *C* results in a softer margin, allowing more misclassifications but potentially achieving better generalization on unseen data. Conversely, a larger value of *C* leads to a harder margin, minimizing misclassifications at the risk of overfitting the training data.^29^

### 3.5 SVM Based Fault Classification System

**Figure 6:**
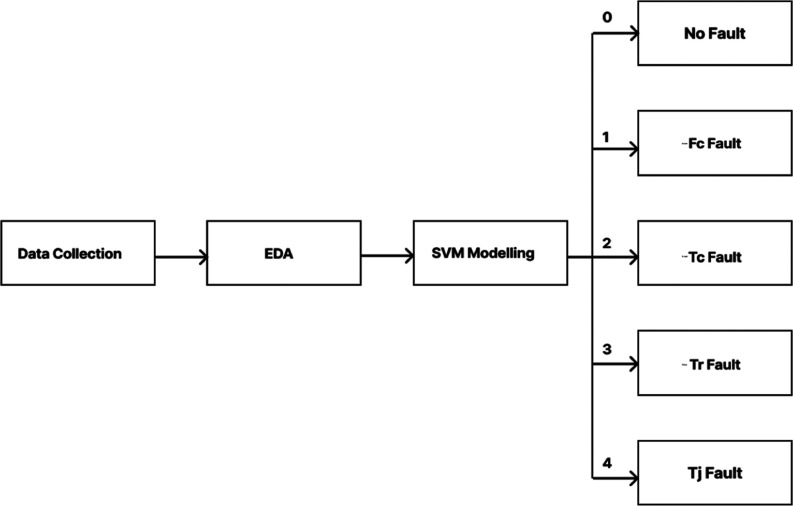
Block diagram of the FD model.

The fault categorization in the BR process is conducted according to the diagram given in Figure 6.

#### 3.5.1 Data Collection

In the BR process, the data is gathered from different sensors. Mainly, actuator data (*F*_c_), temperature of the coolant (*T*_c_), temperature of the reactor (*T*_r_), and jacket temperature (*T*_j_). The recorded data is written to the CSV file. The data gathered is nonlinear in nature, as it was collected from various sensors. Nonlinear data refers to a type of data distribution or relationship between variables that a linear function cannot accurately model or represent. In contrast to linear data, where a straight line can adequately describe the relationship between variables, nonlinear data exhibits more complex patterns that may follow curves, circles, parabolas, or other nonlinear shapes. When dealing with nonlinear data, linear models, such as simple linear regression, may not be sufficient for capturing the underlying patterns, leading to poor predictive performance. In such cases, more advanced models capable of handling nonlinear relationships are often required. SVMs with nonlinear kernel, can effectively capture nonlinear patterns in data from pilot plant BR.^30,31^

#### 3.5.2 Data Preprocessing

**Figure 7:**
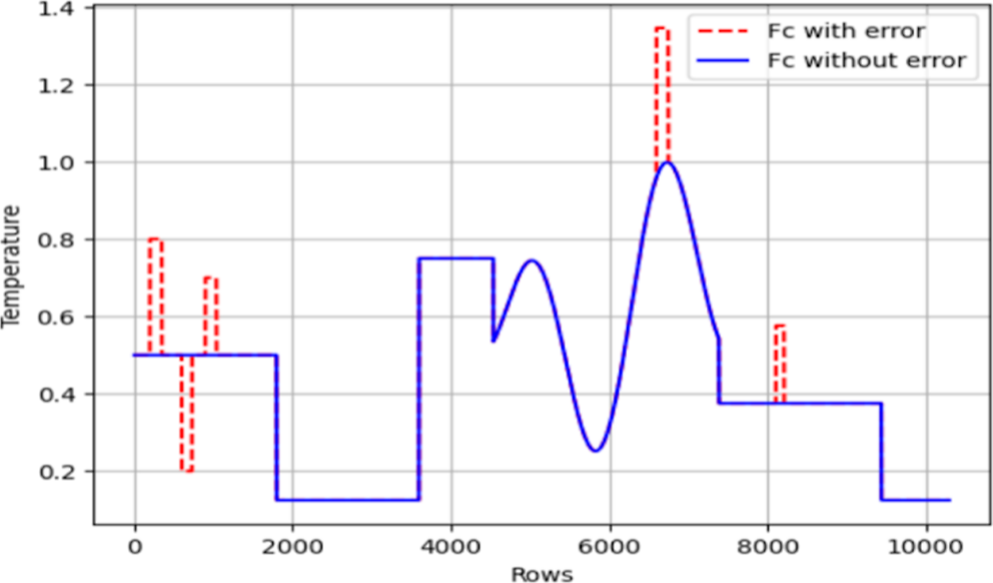
Error introduced *F*_c_ data.

**Figure 8:**
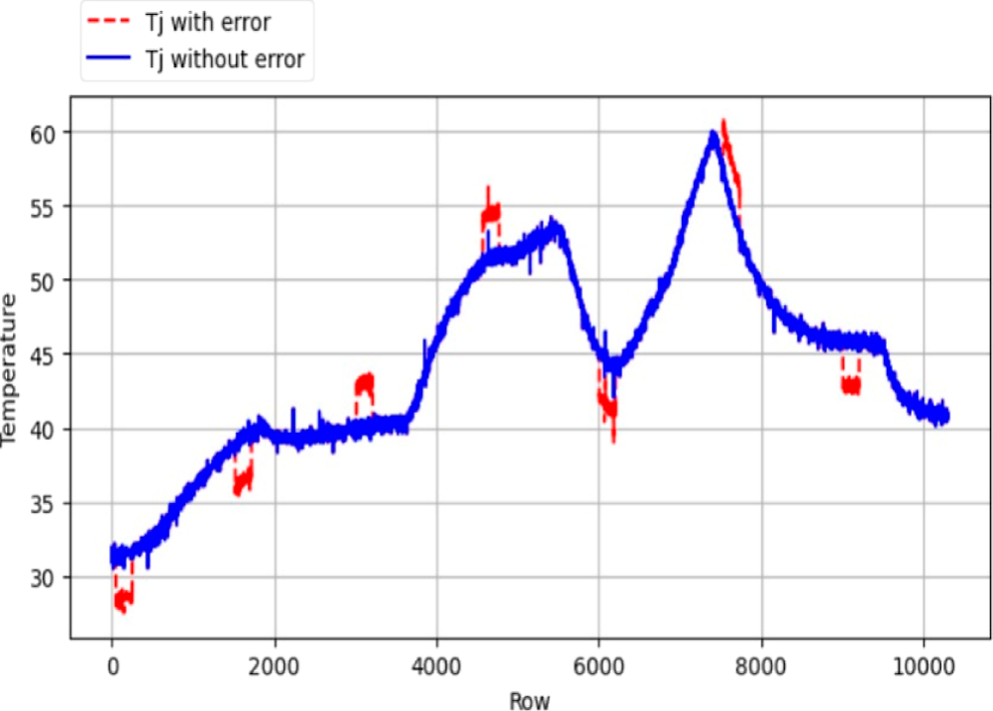
Error introduced *T*_j_ data.

**Figure 9:**
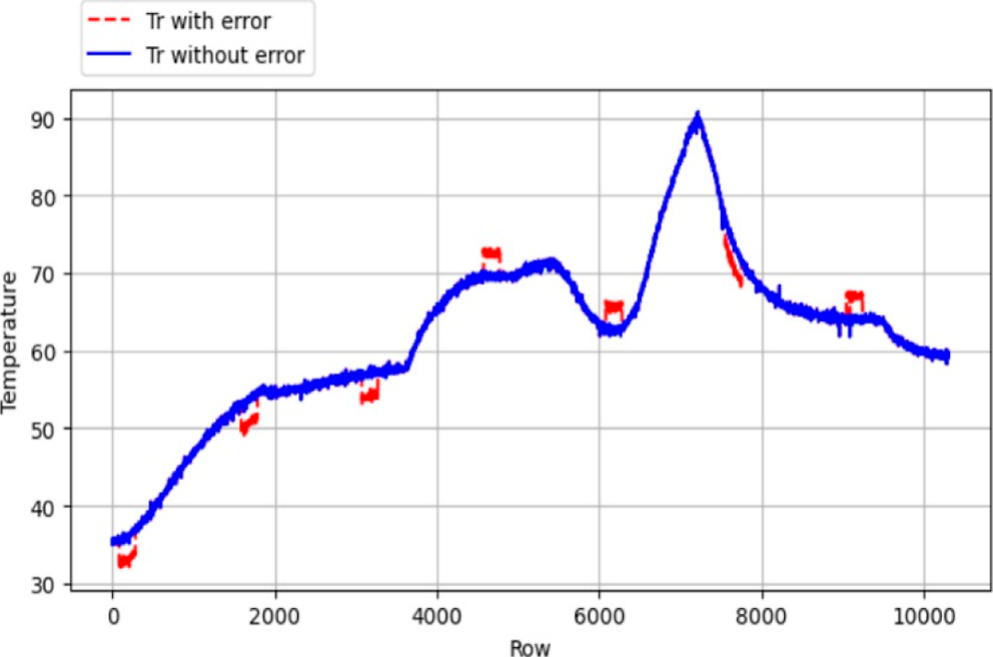
Error introduced *T*_r_ data.

**Figure 10:**
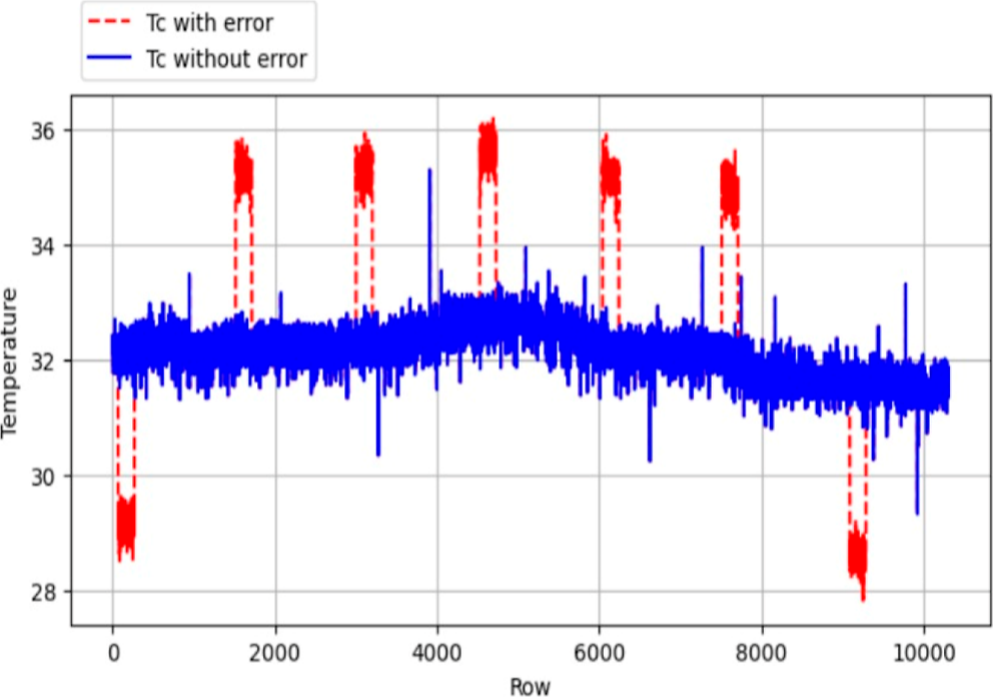
Error introduced *T*_c_ data.

In the data preprocessing phase, we began with a data set containing four features, originally error-free, and a target variable with five classes (0, 1, 2, 3, 4) representing different fault categories (0: no error, 1: error in *F*_c_, 2: error in *T*_c_, 3: error in *T*_r_, 4: error in *T*_j_). Figures 7–10 were employed to visually illustrate and characterize this data set structure, providing insights into the distribution of error classes.

Given the nonlinear nature of the data, we recognized the importance of scaling for effective training of the SVM model. Scaling was employed to ensure that all features were on a similar scale, preventing certain features from dominating others during the training process. The scaled data was then split into training and testing sets, with a test size of 40%, to facilitate model evaluation on unseen data. This rigorous preprocessing methodology aimed to create a reliable foundation for training the SVM model to accurately classify faults in the BR process based on the introduced errors. The systematic approach taken in data preprocessing sets the stage for robust model training and subsequent fault classification.^21^

#### 3.5.3 SVM Model and Hyper Parameters Tuning

After splitting the data into training and testing sets, the GridSearchCV method was used to find the best hyperparameters for the SVM model. A parameter grid was defined to explore various combinations of hyperparameter values, and GridSearchCV efficiently performed cross-validated model training on each combination. GridSearchCV is a powerful technique that automates the hyperparameter tuning process by performing a search over a predefined set of hyperparameter values. In the proposed model, the hyperparameters included *C* (regularization parameter), γ (kernel coefficient for “rbf”), kernel type, and decision function shape. The goal was to find the combination that maximizes the model’s accuracy in fault classification.^22^

By systematically trying various combinations, GridSearchCV helps navigate the hyperparameter space and identify the values that lead to the best model performance. The use of cross-validation ensures that the model’s performance is evaluated across multiple subsets of the training data, providing a more reliable estimate of its generalization capabilities.^22^

Upon completion of the GridSearchCV process, a comprehensive dataframe was constructed, summarizing the hyperparameter configurations and their corresponding accuracies. The sorting of this dataframe based on accuracy values facilitated the identification of the best-performing combination of hyperparameters. The chosen hyperparameter values (*C* = 0.5, γ = 100, kernel = “rbf”, decision_function_shape = “ovo”) produced a model with an impressive accuracy of 98.33%. This shows that the hyperparameter tuning process is an effective way to improve the SVM model for fault classification in the BR process.

**Figure 11:**
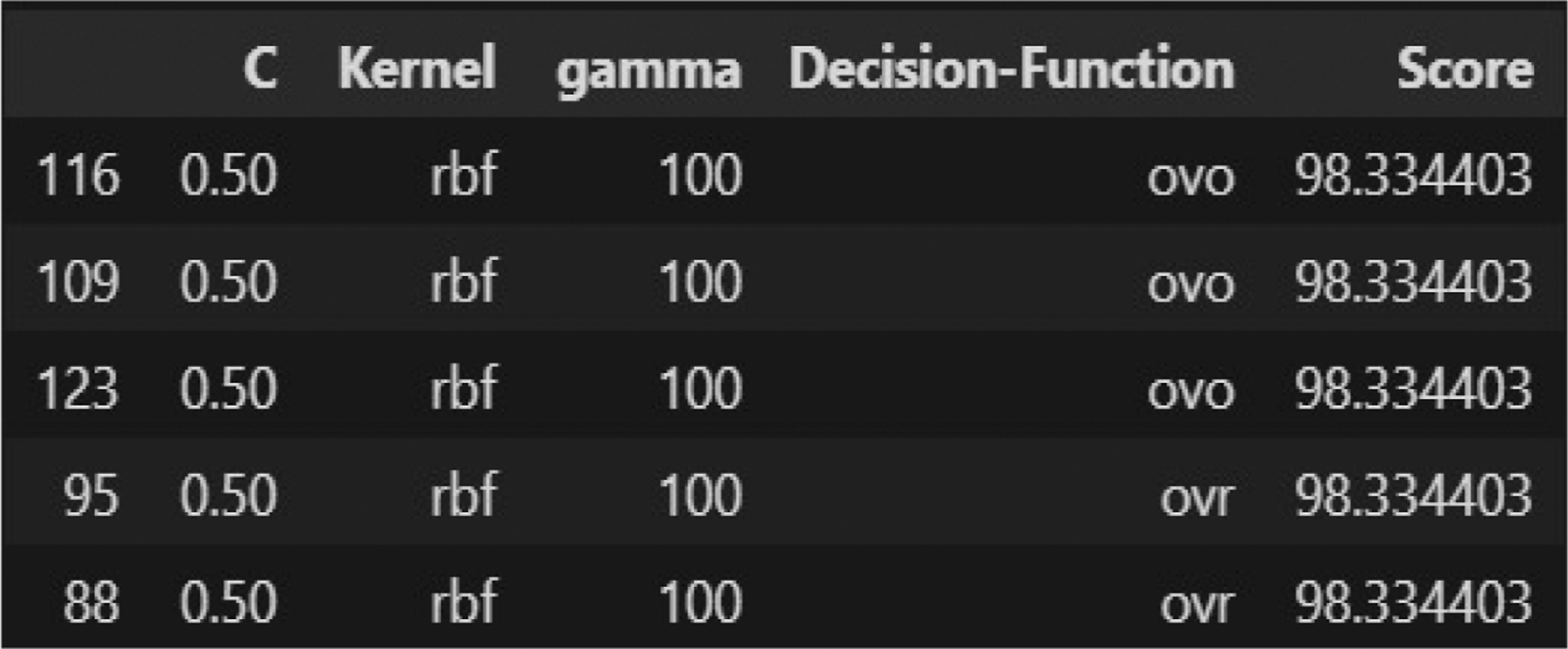
Output of SVM hyperparameters and their corresponding accuracy.

This fine-tuned model is expected to exhibit improved performance on new, unseen data, enhancing its practical utility. After that, the testing data were used to figure out how accurate each SVM model was, and a full dataframe was made with columns for hyperparameters and their corresponding accuracies. The dataframe was subsequently arranged in descending order according to accuracy values, revealing the combination with the highest accuracy, as depicted in Figure 11. This meticulous hyperparameter tuning process ensures that the SVM model is fine-tuned for optimal performance in fault classification, enhancing its reliability and effectiveness in real-world applications.^18^

#### 3.5.4 Classification Visualizations

A dedication to simulating real-world conditions and ensuring model robustness is evident in the deliberate introduction of errors into the data set, meticulous scaling, and thoughtful selection of hyperparameter tuning strategies. The use of SVM with a nonlinear kernel, specifically the RBF, proved instrumental in capturing the intricacies of the nonlinear data, leading to a model capable of accurately classifying faults. GridSearchCV’s hyperparameter tuning was key to finding the best set of hyperparameters. The values chosen (*C* = 0.5, γ = 100, kernel = “rbf”, decision_function_shape = “ovo”) produced an impressive 98.33% accuracy.^22^

**Figure 12:**
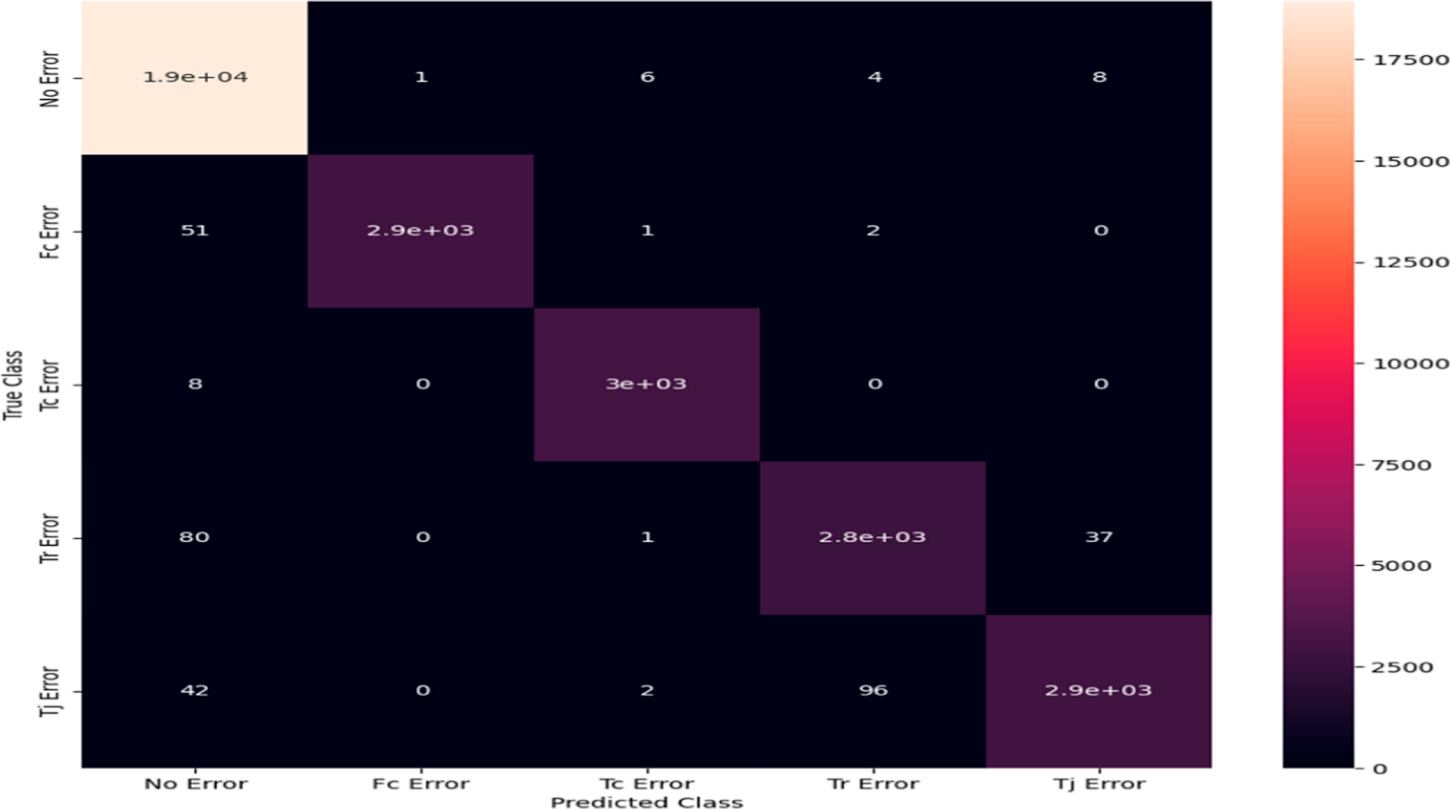
Output of confusion matrix.

The confusion matrix for multikernel SVM using the Gaussian kernel function is depicted in Figure 12. It provides a distinct depiction of how the defects are differentiated from the correct ones. Each column in the confusion matrix represents the number of cases that were predicted to belong to a specific class.^14^

**Figure 13:**
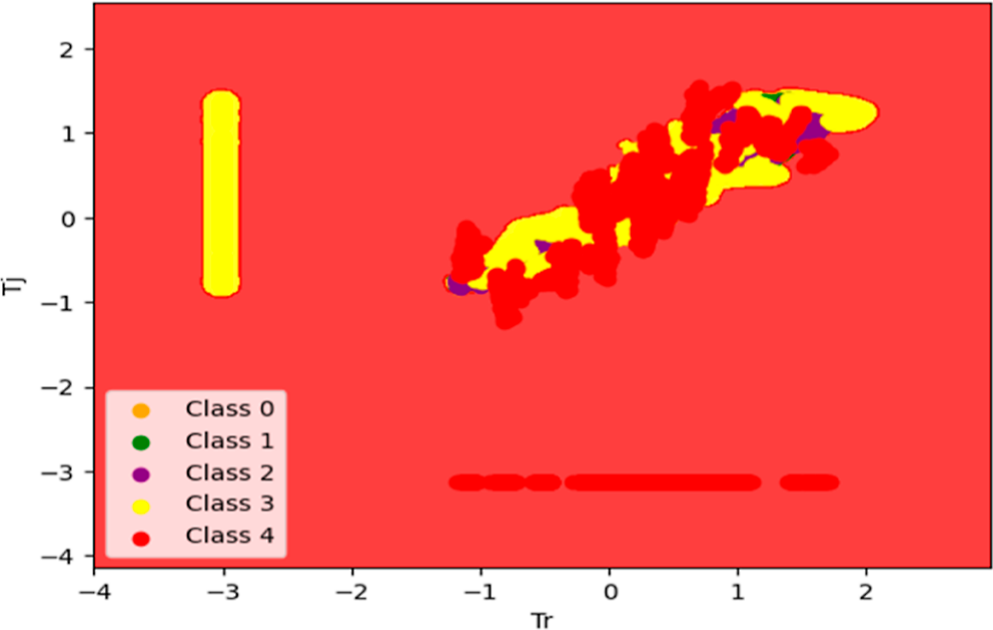
Representation of SVM classification involving *T*_r_ vs *T*_j_.

Here, the SVM model accurately classifies faults in the BR process, specifically focusing on the SVM classification involving *T*_r_ vs *T*_j_. Figure 13 is a 2D representation that provides valuable insights into the separability of these two error classes. There are a total of 19,181 datapoints belonging to the *no error* target class. Out of those datapoints, 19,000 were classified correctly, and others were misclassified.

**Figure 14:**
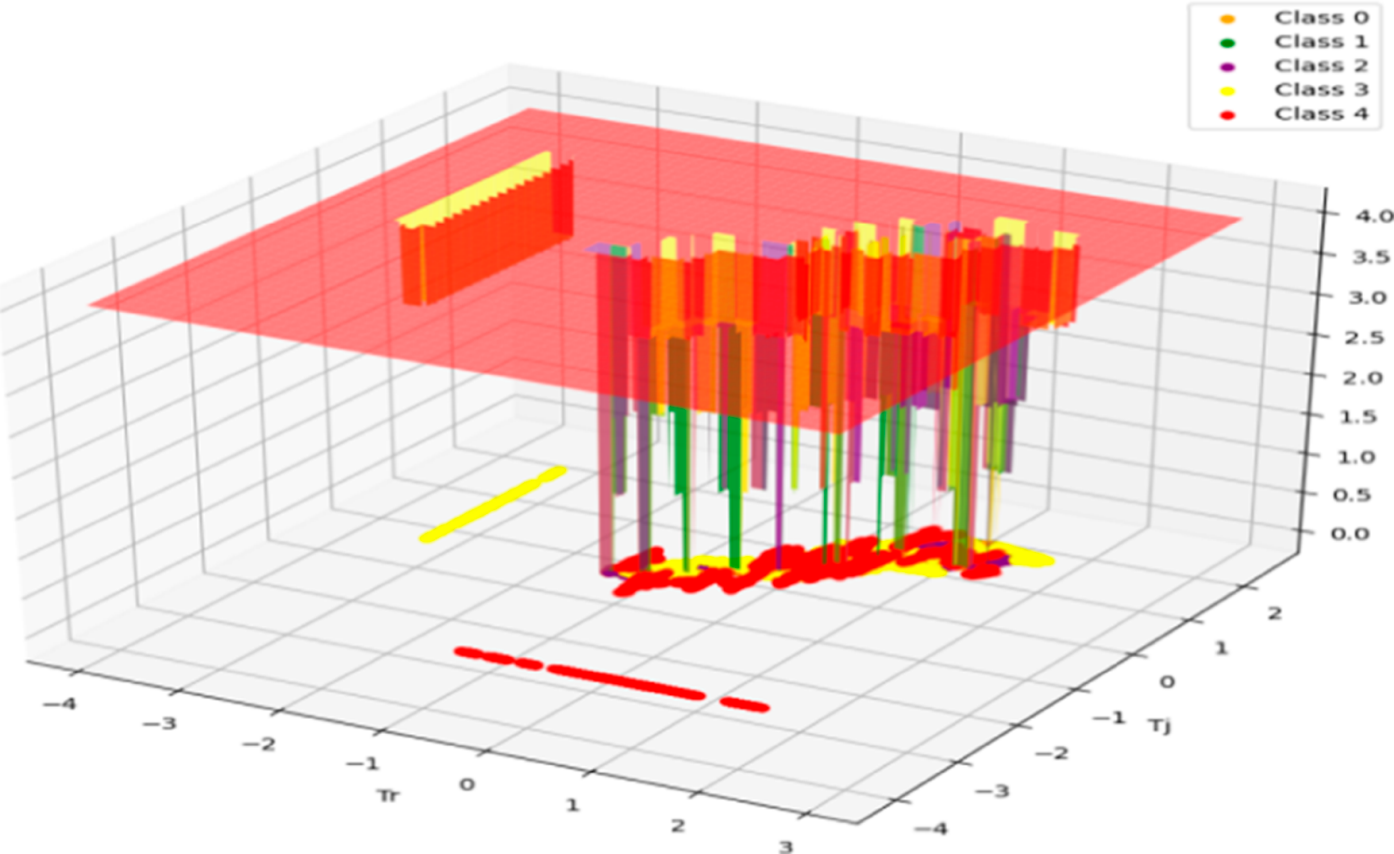
Representation of SVM classification involving *T*_r_ vs *T*_j_.

Figure 14 goes beyond traditional 2D representations, providing a more intricate depiction of the spatial relationships among features and the target variable. In this 3D representation, the model’s decision boundaries and separation between *T*_r_ and *T*_j_ error classes are effectively showcased. This enables a detailed understanding of how the SVM captures the complexity of the data set in three dimensions.

The depicted 3D plot in Figure 14, illustrates the distribution of data points, with the *x* and *y* axes corresponding to the features “Temperature Reactor” and “Jacket Temperature”, respectively. The *z*-axis serves to assign each data point to one of the five classes present in the data set. Notably, the red-colored plane delineates the data points accurately classified as belonging to the “*T*_j_ Error” class, exemplifying the model’s precision in this classification. Conversely, the yellow class represents instances classified as the “*T*_r_ Error”, highlighting a notable misclassification trend within this category. The majority of data points pertaining to both the “Temperature Reactor” and “Jacket Temperature” features are correctly classified, underscoring the efficacy of the model in discerning patterns within these variables. However, misclassifications are observed within the green, purple, and orange regions, signifying areas where the model’s performance may be improved. This comprehensive visualization offers insights into the model’s classification accuracy and areas for potential refinement.^29,32,33^

## 4 Results and Conclusions

The fault classification using nonlinear SVM for the BR process was implemented using GridSearchCV’s hyperparameter tuning approach to finding the best set of hyperparameters. The values chosen (*C* = 0.5, γ = 100, kernel = “rbf”, decision_function_shape = “ovo”) produced an impressive 98.33% accuracy. This paper has inferred from the data that multiclass nonlinear kernels are well suited for this model. Random generation of faults in data has been introduced at time-scale interval points with labeling of the error in the predictive features. Table 2 shows the model performance evaluation in terms of precision, recall, and F1-score over each target class. The performance of the proposed model in terms of the defined parameters is at least 96% over each target class. Table 3 shows the model performance evaluation in terms of accuracy, precision, recall, and F1-score over each kernel. The performance of the proposed rbf kernel shows better performance in terms of defined parameters over other kernel methods.

**Table 2:** Model Performance Evaluation Over Each Target Class

	precision	recall	F1-score
no error	0.96	0.98	0.98
*F*_c_ error	0.99	0.99	0.99
*T*_c_ error	1.00	1.00	1.00
*T*_r_ error	0.97	0.97	0.97
*T*_j_ error	0.99	0.97	0.98

**Table 3:** Model Performance Evaluation Over Each Kernel

Kernel	accuracy	precision	recall	F1-score
linear	44.27	72.91	44.26	46.24
poly	75.56	78.50	75.56	75.80
sigmoid	44.49	57.87	44.49	43.40
Gaussian (RBF)	98.90	97.70	97.64	97.65

## 5 Future Work

Integration of the proposed SVM model for fault classification is yet to be tested on the pilot plant BR setup along with the closed loop RNN-NMPC and DNN-NMPC for trajectory tracking. Since the data is in a time series format, this work has yet to be tested with the deep learning model and verify its performance. The verification of finding other random generated fault is to be considered while doing real time experimentation. Downloading the developed SVM Python code into Jetson Orin 8GB board is also in progress for closed loop experimental validation for online fault classification.
